# Standardization of the surgical technique and reporting for radical right colectomy with central vascular ligation and complete mesocolic excision (RRoC-STAR): Delphi consensus

**DOI:** 10.1093/bjsopen/zraf066

**Published:** 2025-06-12

**Authors:** Giuseppe S Sica, Gabriele Anania, Cristina Fiorani, Leandro Siragusa, Danilo Vinci, Marco Caricato, Paolo Delrio, Antonino Agrusa, Gianandrea Baldazzi, Rossella Reddavid, Gianluca Pellino, RRoC-STAR Collaborative Group, A Garcia-Granero, L Ferraro, K B Lygre, V Balaban, P T Sugoor, G Tzovaras, W T-L Chen, B S Min, M N A Tan, T Sammour, A Takashi, S Benz, V Ozben, C Pedrazzani, G Formisano, S H Kim, C Akyol, N West, A M Guida, A Divizia, A Giani, G S Banipal, I al-Najami, W Luo, V Bellato, B M Pirozzi, I Seow-En, R Bernhoff, G Dimofte, A M Musina, T Konishi, Y S Lee, S Morarasu, N Gouvas, T Zurleni, P Carnevali, Y Fukunaga, M Pramateftakis, Z Balciscueta, M Ali Chaouch, G J Chang, S Di Carlo, İ Hamzaoglu, G Di Buono, A Petrov, K Mathis, A M Kazaryan, M Mazzola, S Buscemi, E Jovine, S Scabini, E Bertani, G Ferrari, J Khan, F Pfeffer, G Spinoglio, G Luglio, G Navarra, H Kobayashi, S Merkel, D Cassini, G N Piozzi, M Spalluto

**Affiliations:** Minimally Invasive and Gastrointestinal Surgery Unit, University of Rome Tor Vergata, Rome, Italy; Department of Medical Science, University of Ferrara, Ferrara, Italy; Minimally Invasive and Gastrointestinal Surgery Unit, University of Rome Tor Vergata, Rome, Italy; Division of Colon and Rectal Surgery, IRCCS Humanitas Research Hospital, Rozzano, Milan, Italy; Department of Surgical Science, Policlinico Tor Vergata—University Tor Vergata, Rome, Italy; Colorectal Surgery Unit, Università Campus Bio-Medico di Roma, Rome, Italy; Colorectal Surgical Oncology, Istituto per lo studio e la cura dei tumori ‘Fondazione Giovanni Pascale IRCCS’, Naples, Italy; Department of Surgical, Oncological and Oral Sciences, University of Palermo, Palermo, Italy; ASST Ovest Milanese, P.O. Nuovo Ospedale di Legnano, Legnano, Italy; Department of Oncology, San Luigi University Hospital, University of Turin, Turin, Italy; Vall d’Hebron University Hospital, Universitat Autonoma de Barcelona UAB, Barcelona, Spain; Department of Advanced Medical and Surgical Sciences, Università degli Studi della Campania ‘Luigi Vanvitelli’, Naples, Italy

## Abstract

**Background:**

Complete mesocolic excision refers to a radical right hemicolectomy for cancer following embryologically defined anatomical planes. However, heterogeneity in definitions and techniques is a barrier to research. The aim of the Radical Right Colectomy—Surgical Technique Approved Report (RRoC-STAR) collaborative is to provide international expert consensus-based definitions and standardized terminology and surgical steps for right hemicolectomy for locally advanced colon cancer.

**Methods:**

Authors of publications reporting on radical right hemicolectomy techniques were invited to complete an ACCORD-compliant Delphi questionnaire (two rounds). A standardized name (for the procedure) and a data sheet for reporting the procedure were proposed, along with 21 items, including terminology and surgical steps. The assembled panel was asked to vote for each item, with consensus considered to have been reached for items that achieved at least 80% agreement.

**Results:**

Of 162 invited authors, 67 completed both Delphi rounds. All but 1 of the 21 items received consensus after 2 rounds. Consensus was reached on the use of the proposed data sheet for reporting, the term radical right colectomy (RRC), and the surgical steps deemed necessary for RRC, namely preservation of mesocolic integrity, sharp dissection of the anterolateral surface of the superior mesenteric vein up to the middle colic vein, ligation at the origin of vessels, and dissection of lymphoadipose tissue around the gastrocolic trunk of Henle.

**Conclusion:**

This study provides an international expert consensus-based definition and standardization of terminology and the surgical steps required to perform RRC. A comprehensive data sheet for reporting RRC is introduced to enable data homogenization from current and future studies.

## Teaser

This study provides an international expert consensus-based definition and standardization of terminology and the surgical steps required to perform radical right colectomy with complete mesocolic excision, central vascular ligation, and D3 lymphadenectomy. A comprehensive data sheet for reporting radical right colectomy is introduced to enable data homogenization from current and future studies.

## Introduction

Surgery for locally advanced colon cancers requires wide resection of the bowel segment involved and its lymphatic drainage. *En bloc* colonic and mesenteric resection is recommended to define cancer stage and to identify and remove potential lymph node metastases^1^. European Society For Medical Oncology and American Society of Clinical Oncology guidelines recommend that at least 12 lymph nodes should be removed when feasible^2,3^. Several authors^4,5^ have suggested improved overall survival and disease-free survival in patients with mesocolic lymph node involvement who undergo a more extensive lymphadenectomy, such as the D3 lymphadenectomy advocated by the Japanese Society for Cancer of the Colon and Rectum^6^.

In the late 1980s and early 1990s, Heald^7^ highlighted the importance of a precise surgical dissection along embryologically designed anatomical planes in rectal cancer surgery to achieve total mesorectal excision. In 2009, the German surgeon Werner Hohenberger^8^ applied the principles of total mesorectal excision to right hemicolectomy for cancer. He introduced and popularized the idea of complete mesocolic excision (CME), emphasizing the importance of preserving mesocolic integrity and ensuring its complete removal also in colon cancer surgery. Applying the principle of a meticulous sharp dissection following the embryological surgical plane offered by the fusion of the fascia of Toldt and the fascia of Fredet to dissect the right mesocolon from the retroperitoneum, Hohenberger reported a significant reduction in the 5-year recurrence rate at his institution from 6.5 to 3.6%^8^.

Following the description of CME by Hohenberger, the interest in extensive dissection based on embryological principles in colon cancer has resulted in the publication of at least two meta-analyses on this topic^9,10^. However, due to the frequent anatomical variations in the vascular anatomy of the right colon, differences in the localization of the neoplasia, and differences in surgical techniques, there has been no standardization of the technique, contributing to difficulties comparing outcomes across studies. A recent systematic review published by Sica *et al*.^11^ highlighted the significant heterogeneity in both the definitions of and the surgical steps required to obtain a right hemicolectomy with CME, central vascular ligation (CVL), and D3 lymphadenectomy. This lack of uniformity leads to challenges in interpreting study outcomes, limits generalizability, and hinders effective comparisons.

The present Delphi consensus addresses the need for a standardized definition of radical right hemicolectomy for cancer with CME, CVL, and D3 lymphadenectomy, reviewing the concerns regarding definitions, the quality of reporting, and surgical techniques. The aim of this consensus was to produce a standardized data sheet for reporting this operation and a comprehensive definition of the surgical steps involved. The final aim was to ensure consistency, reliability, and reproducibility to enable comparisons of results and outcomes.

## Methods

A Delphi consensus with the Delphi method^12^ was undertaken using a structured, reiterating questionnaire sent to a group of corresponding, first, or last authors from the 99 articles included in a previous systematic literature search^13^. An up-to-date systematic literature search was conducted to invite authors of papers published after the earlier review (up to August 2023) to participate in the scoping survey.

The following databases were searched: MEDLINE (PubMed), ClinicalTrials.gov, and Cochrane Database (last search 31 August 2023). The following search terms were used: complete mesocolon excision, D3, central vascular ligation, right hemicolectomy, and colon cancer. Cross-searches were performed for references from retrieved articles. Records were screened for relevance based on their title and abstract, after which the full text of identified articles was analysed. The types of studies eligible for inclusion were original articles, systematic reviews, and meta-analysis. A fundamental inclusion criterion was the presence of a clear definition of the surgical technique in the methods section. Two authors (L.S. and C.F.) independently screened each record from full-text articles for eligibility and extracted the data. Disagreement was resolved by discussion and consensus; if no agreement was reached, the lead author (G.S.S.) was consulted. The systematic literature review was performed following the current PRISMA 2020 guidelines for systematic reviews^13^. The database search identified 188 articles. After initial screening, the exclusion of duplicates, and full-text review, a further 62 articles were identified and included in the qualitative review. The systematic search processes are summarized in *Figs S1* and *S2*.

Authors were invited to take part in the Radical Right Colectomy—Surgical Technique Approved Report (RRoC-STAR) Delphi consensus. The panellists voted in two rounds, between December 2023 and August 2024, on a standardized definition of RRC, how to report the operation, definitions of the surgical steps, and which of those steps are necessary to achieve RRC. The Delphi consensus process was endorsed by the Italian Society of Surgical Endoscopy and followed the CREDES guidelines from the EQUATOR Network^14^ and the reporting of items as per the ACCORD project protocol^15^.

### Delphi process overview

The Delphi consensus was designed by the RRoC-STAR steering committee, which created a proposed definition for a right hemicolectomy for cancer with CME, CVL, and extended (D3) lymphadenectomy. The proposal was based on a review of current nomenclature, the identification of overlaps and differences in the terminology, and surgical steps reported in the relevant literature that were pertinent to the procedure.

An *a priori* consensus standard was set at > 80% for full agreement or partial agreement with a statement, which was considered a majority positive verdict^16^.

The survey was designed using Google Forms (Google LLC, Menlo Park, CA, USA).

**Fig. 1:**
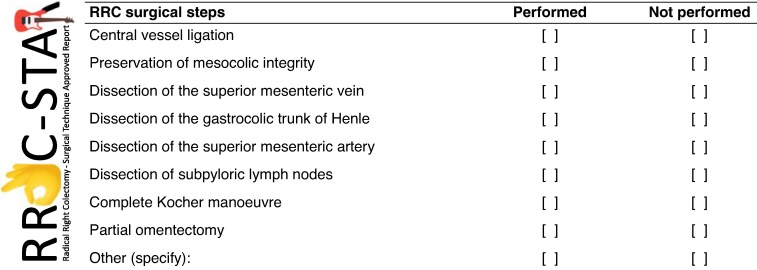
RRoC-STAR data sheet to report RRC surgical steps RRoC-STAR, Radical Right Colectomy—Surgical Technique Approved Report; RRC, radical right colectomy.

The purpose of this Delphi consensus was explained to all participants. First, participants were asked to vote on the definition proposed by the steering committee, namely ‘Radical right colectomy (RRC) for a right hemicolectomy for cancer with CVL, CME, and extensive (D3) lymph node dissection’. The consensus group was then asked to assess the RRoC-STAR data sheet for standardizing the reporting of RRC (*Fig. 1*), as well as three domains, including 21 items, as follows: Domain 1 (Items 1–4), how to report; Domain 2 (Items 5–12), definitions of all the surgical steps related to the operation; and Domain 3 (Items 13–21), the surgical steps deemed necessary to achieve RRC. At the end of each domain, space was provided for feedback on any disagreements and for the consensus rating. The survey structure is summarized in *Table S1*.

All participants were informed that results of the survey would be used for further statistical evaluation and scientific publication. Anonymity was guaranteed by the study design, and authorship as part of the collaborative group was voluntary.

A dedicated account was created to deliver the survey, answer query-related inconsistencies in survey responses, and confirm authorship.

A pilot version of the consensus was tested among steering committee members between 10 and 20 November 2023; the first Delphi round was launched on 1 December 2023 and closed on 29 February 2024.

On 1 March 2024, the data set from the first round was extracted and analysed by the steering committee. Items that reached consensus in the first round were not subjected to further voting, unless revised. Items with < 80% agreement were carefully evaluated and revised according to the comments received before being subjected to further voting. If relevant comments or corrections were received for items with > 80% agreement in the first round, these items were also revised and subjected to voting in the second round.

The second Delphi round was launched on 10 April 2024 and closed on 31 August 2024.

Participants were informed of the consensus obtained in the previous round before the second round of voting. This iterative process aimed to achieve increasing consensus. To maintain the anonymity of the process, experts were not aware of the responses of individual participants, only those of the whole group.

Only answers of participants who completed both Delphi rounds were considered in the final data analysis.

By October 2024, the full data set of the second Delphi round was extracted from Google Forms, incomplete responses and duplicates were removed and the final database was sent for result analysis.

### Statistical analysis

Each round generated data expressed as the percentage agreement for each item.

## Results

The Delphi consensus process took place between November 2023 and August 2024. In all, 162 authors were invited to take part in the voting panel; 69 (42.6%) completed the first round of voting on the standardized definition, data sheet, and 3 domains with a total of 21 items. Of the 21 items, agreement of > 80% was reached for 12 items (57.1%); 2 of these items were amended based on relevant feedback and resubmitted for reconsideration. In the second round, 67 panellists (97.1%) voted, with consensus reached for all but 1 of the 21 items. The item for which agreement was not reached was omentectomy in Domain 3 (surgical steps required); because of the considerable disparity in comments from the panellists and the fact that omentectomy was rarely reported in the papers examined, it was removed. After the two rounds of voting, consensus was reached for a standardized definition, reporting data sheet, and 20 statements. Following the first round, consensus was reached on RRC as the proposed surgical procedure definition (82%) and that all authors investigating RRC should report on the surgical steps deemed necessary and the quality of the specimen (98.5%).

The required surgical steps for a technically correct RRC that obtained consensus agreement are as follows: central vessel ligation, defined as ligation at the roots of the ileocolic vessels, right colic vessels, superior right colic vein (when present), and the right branch of the middle colic vessels (95.6%); preservation of mesocolic integrity, defined as dissection along the embryological plane and complete excision of the mesocolon, conserving the integrity of its anterior and posterior sheaths and the mesenteric sail (98.5%); dissection of the superior mesenteric vein (SMV), defined as dissection along the anterior and lateral right face of the SMV up to the origin of the middle colic vein/Henle’s trunk (98.5%); and dissection of the gastrocolic trunk of Henle, defined as dissection along the common gastrocolic trunk of Henle (84.1%).

The surgical steps that are not required are: dissection of the superior mesenteric artery (SMA), defined as surgical dissection of the anterior and lateral right face of the SMA (86.6%); dissection of the subpyloric lymph nodes, defined as removal of lymphoadipose tissue around the origin of the gastroepiploic artery (83.6%); ligation of the gastroepiploic vessels (91%); and complete Kocher manoeuvre, defined as complete mobilization of the primary to tertiary portions of the duodenum to access the retropancreatic and caval lymph nodes (92.6%) (*Table 1*).

**Table 1:** Summary of RRoC-STAR Delphi consensus statements

Statement	Agreement (%)	Round
**Terminology, how to report RRC, and RRoC-STAR data sheet contents**		
Statement 1. A consensus on the right terminology to be used to describe a radical right colectomy for cancer is necessary. The proposed term is RRC (radical right colectomy).	82.0	1
Statement 2. All authors investigating RRC should report defined surgical steps.	98.5	1
Statement 3. All authors performing RRC should report on the quality of the specimen, and preferably produce photographic and/or histopathological evidence of the specimen. The proposed classification for the quality of the specimen is that described by Benz *et al*.^18^. The proposed data sheet to classify surgical specimens is that described by Garcia-Granero *et al*.^19^.	98.5	2
Statement 4. The RRoC-STAR data sheet should be used to report RRC. Consistent reporting of procedures is needed to obtain reliable conclusions in present and future trials.	89.8	1
**Definitions of RRC surgical steps**		
Statement 5. Central vessel ligation is defined as ligation at the roots of the ileocolic vessels, right colic vessels, superior right colic vein (when present), and the right branch of the middle colic vessels.	97.0	2
Statement 6. Preservation of mesocolic integrity is defined as dissection along the embryological plane and complete excision of the mesocolon, conserving the integrity of its anterior and posterior sheaths and the mesenteric sail.	95.6	1
Statement 7. Dissection of the SMV is defined as dissection along the anterior and lateral right face of the SMV up to the origin of the middle colic vein/Henle’s trunk.	88.4	1
Statement 8. Dissection of the SMA is defined as surgical dissection of the anterior and lateral right face of the SMA.	89.6	2
Statement 9. Dissection of the gastrocolic Henle’s trunk is defined as dissection along the common gastrocolic Henle’s trunk. Pancreatic and gastroepiploic veins should not be divided, unless necessary.	91.3	1
Statement 10. Dissection of subpyloric lymph nodes is defined as removal of lymphoadipose tissue around the origin of the gastroepiploic artery.	92.5	2
Statement 11. Complete Kocher manoeuvre is defined as complete mobilization of the first to third portions of the duodenum to access retropancreatic and caval lymph nodes.	83.6	2
Statement 12. Partial omentectomy is defined as omental division and resection of the right half of the entire greater omentum.	97.0	2
**Steps required for RRC**		
Statement 13. Central vessel ligation should be considered an integral part of RRC.	95.6	1
Statement 14. Preservation of mesocolic integrity should be considered an integral part of RRC.	98.5	1
Statement 15. Dissection of the SMV should be considered an integral part of RRC.	98.5	1
Statement 16. Dissection of the GCTH should be considered an integral part of RRC.	84.1	1
Statement 17. Dissection of the SMA is not required for RRC.	86.6	2
Statement 18. Dissection of subpyloric lymph nodes is not required for RRC.	83.6	2
Statement 19. Ligation of gastroepiploic vessels is not required for RRC.	91.0	2
Statement 20. Complete Kocher manoeuvre is not required for RRC.	92.6	2

RRoC-STAR, Radical Right Colectomy—Surgical Technique Approved Report; RRC, radical right colectomy; SMV, superior mesenteric vein; SMA, superior mesenteric artery, GCTH, gastrocolic trunk of Henle.

## Discussion

Since the introduction in 2009 by Werner Hohenberger of CME in colon cancer surgery^8^, several studies and randomized clinical trials^17–19^ have been published to assess and validate the surgical technique. There is significant heterogeneity and consistent overlap in definitions and surgical steps, with current terminology using several acronyms, such as CME, D3, CME + CVL, CVL, CME + D3, and mCME (modified CME)^11^. This lack of uniformity poses challenges in interpreting study outcomes, limits generalizability, and hinders effective comparisons. A Delphi consensus on the indications for CME in right hemicolectomy, surgical training, and performance assessment has recently been published^20^. In that study, consensus required the agreement of 70% of participants (38 surgeons) on the need to perform CVL, preservation of mesocolic integrity, and dissection along the SMV when referring to CME.

The present Delphi consensus, the inclusion criteria for surgeon experts were stricter, including only those authors who have previously published on this specific topic detailing the surgical technique, and consensus was set at agreement of > 80% of participants. A simple and inclusive nomenclature was chosen and agreed upon, and the steps required for RRC to be performed were clearly identified.

A standardized data sheet with all the reported surgical steps was developed to highlight differences in surgical technique. The data sheet was designed to be simple, with tick boxes for an effective and reproducible way of reporting RRC.

Regarding specimen quality, the panellists agreed on using the classification of Benz *et al*.^21^ for specimen quality and the classification of Garcia-Granero *et al*.^22^ for histopathological analysis. In the prospective trial of Benz *et al*.^21^, photographs of right hemicolectomies were taken from 38 hospitals in Germany with the aim of defining the quality of CME specimens, as well as the quality and extension of lymph node dissection. The panel, for reasons of organization and opportunity, did not consider it necessary to routinely obtain photographic evidence of specimen quality, but in all cases the mesentery should be laid out flat, without external tension, and distances from the tumour to the vascular ties measured.

Given the wide variability in RRC descriptions in the literature, the RRoC-STAR data sheet could help address these limitations by systematically capturing critical procedural details, resulting in more reliable evidence synthesis and clearer recommendations. Furthermore, standardized reporting according to agreed guidelines or checklists (for example, the tumour node metastasis (TNM) staging system or Clavien–Dindo classification) is crucial to reduce variability and inconsistencies between different centres and surgical teams^23,24^.

Since its introduction in 2009, CME has been based on two key surgical principles: dissection along embryological planes and high vascular ties^8^. Both techniques maximize the number of harvested lymph nodes, which is linked to improved survival and oncological outcomes, because the number of dissected lymph nodes is a potential positive prognostic factor^25,26^. Colon cancer spreads from pericolic lymph nodes to lymph nodes along the arterial arcade, and then centrally to the root of the vessels. However, extended lymphadenectomy, dissecting along the lateral and anterior border of the SMA, may also increase the surgical risk; in most institutions, the SMV is used as a landmark for an adequate lymphadenectomy. In doing this, following the guidelines of the Japanese Society of Colon and Rectal Surgeons, stations 203, 213 and 223 are removed^27^.

The recently published RELARC trial^28^ failed to find evidence of superior disease-free survival outcomes for more extensive lymphadenectomy compared with standard D2 lymph node dissection in primary surgical excision of right-sided colon cancer. The authors concluded that standard D2 dissection should be the routine procedure for these patients, and that CME with extensive lymphadenectomy should only be considered in patients with obvious mesocolic lymph node involvement^28^. Interim results from the CoME-in trial^17^, another superiority phase III randomized trial on the same subject, reported that the quality of surgery and the lymph node yield are higher after CME, and recommended continuation of patient recruitment and implementation for optimal comparison. The RESECTAT trial^29^ reported better overall survival of patients with stage III colon cancer on unplanned exploratory analysis. Although of uncertain significance, the debate remains regarding the need for better-quality oncological surgery for colon cancer.

When surgical dissection is performed following rigorous principles of embryologically determined surgical planes, the preservation of mesocolic integrity is thought to improve oncological outcomes by reducing cancer cell seeding and removing skip or in-transit metastases. A correct surgical plane for RRC is one that follows anatomical landmarks. Recognition of the fusion fascia of Fredet allows proper dissection of the surgical trunk of Gillot and the lymphoadipose tissue covering the head of the pancreas, thus exposing and allowing dissection of the gastrocolic trunk of Henle^22,30^.

The results from the present consensus are that SMA dissection should not be routinely performed. The prevailing belief is that SMA dissection poses more risks than benefits^20,31^. Further investigations, examining pros and cons, including overall oncological outcomes are needed.

Infrapyloric lymph nodes (No. 206) are found along the proximal part of the right gastroepiploic artery and vein. The need for routine *versus* selective dissection of these lymph nodes is debated. However, dissecting subpyloric lymph nodes and removing tissue around the origin of the gastroepiploic artery could be a challenging procedure during right colectomy. Nevertheless, a systematic review by Piozzi *et al*.^32^ showed that the incidence of infrapyloric and gastroepiploic lymph node metastases ranges from 0.7 to 22%, suggesting that dissection should be tailored based on tumour location and characteristics, rather than performed routinely during RRC. Given the varying pathways of metastasis, the potential for increased complications, and the uncertain oncological benefits, the consensus in the present study was that routine dissection of subpyloric lymph nodes is not required when performing RRC.

The Kocher manoeuvre, traditionally the first step in pancreatoduodenectomy and gastrectomy, was described by Hohenberger^8^ as a key surgical step during open right hemicolectomy with CME, using a lateral-to-medial approach. The lymph nodes of this region do not drain lymphatic flow from the right colon and, if involved, they should be regarded as distant metastasis; thus, full mobilization of the duodenum is not necessary during laparoscopic or robotic RRC, but it can be helpful in open surgery to ensure correct identification of the fusion fascia of Fredet.

There is a reasonable rationale for omentectomy or partial omentectomy, especially in tumours of the hepatic flexure and transverse colon. However, its embryological origin from the foregut explains why the omentum can be preserved during right hemicolectomy, unless directly involved by the tumour^33,34^. Occasionally omentectomy may be technically necessary due to the frequent fusion of the planes between the mesocolon and omentum, or in case of vascular damage during the dissection. Partial omentectomy has been included in the data sheet to clarify the need for this step in future studies, but there was no consensus regarding the need for this step to achieve RRC.

This consensus selection process, while aiming for objectivity, may have been influenced by inherent biases. This risk was mitigated to some extent by selecting only corresponding, first, or last authors who have published on the terminology and techniques for RRC, ensuring a degree of expertise and experience. This has led to the exclusion of several CME expert surgeons who have not been selected through the systematic literature search or who did not respond to the invitation to be part of the consensus panel. The involvement of steering committee members in data collection could have potentially introduced subjectivity. Their interpretations and decisions during data collection could inadvertently dilute or alter the original intent of the study, potentially leading to unintended biases in the final consensus.

The study’s reliance on an online format, although convenient for participants with busy schedules and different geographical locations, presented certain limitations. The lack of in-person or even virtual meetings to discuss and resolve discrepancies could have hindered effective communication and the resolution of complex issues. Time zone differences and the varying availability of participants may have limited the depth of discussion and the opportunity to fully address dissenting viewpoints. However, the online format did offer the advantage of anonymity for respondents, which may have encouraged more honest and unbiased responses. Finally, the high threshold of 80% agreement for consensus, although ensuring a robust level of agreement among experts, could also be viewed as a limitation. This stringent criterion may have inadvertently excluded valuable minority viewpoints.

## Collaborators

A. Garcia-Granero (Hospital Son Espases, Mallorca, Spain); L. Ferraro (ASST Santi Paolo e Carlo, Milan, Italy); K. B. Lygre (Haraldsplass Deaconess Hospital and Haukeland University Hospital, Bergen, Norway); V. Balaban (Sechenov University, Moscow, Russia); P. T. Sugoor (Kidwai Memorial Institute of Oncology, Bengaluru, India); G. Tzovaras (University of Thessaly, Volos, Greece); W. T.-L. Chen (China Medical University Hsinchu Hospital, Zhubei, Taiwan); B. S. Min (Yonsei University Health System, Seoul, Korea); M. N. A. Tan (Tan Tock Seng Hospital, Singapore); T. Sammour (Royal Adelaide Hospital, Adelaide, Australia); A. Takashi (Cancer Institute Hospital, Japanese Foundation for Cancer Research, Tokyo, Japan); S. Benz (Kliniken Böblingen, Böblingen, Germany); V. Ozben (Acibadem Mehmet Ali Aydinlar University, Istanbul, Turkey); C. Pedrazzani (University of Verona, Verona, Italy); G. Formisano (University of Milan, ASST Santi Paolo e Carlo, Milan, Italy); S. H. Kim (Universiti Malaya, Kuala Lumpur, Malaysia); C. Akyol (Ankara University School Medicine, Ankara, Turkey); N. West (University of Leeds, Leeds, UK); A. M. Guida, A. Divizia (Policlinico Roma Tor Vergata, Rome, Italy); A. Giani (ASST Grande Ospedale Metropolitano Niguarda, Milan, Italy); G. S. Banipal (Akershus University Hospital, Oslo, Norway); I. al-Najami (Odense University Hospital, Odense, Denmark); W. Luo (Suining Central Hospital, Suining, China); V. Bellato, B. M. Pirozzi (University of Rome Tor Vergata, Rome, Italy); I. Seow-En (Singapore General Hospital, Singapore); R. Bernhoff (Capio Saint Göran’s Hospital, Stockholm, Sweden); G. Dimofte, A. M. Musina (Grigore T. Popa University of Medicine and Pharmacy, Iași, Romania); T. Konishi (The University of Texas, M. D. Anderson Cancer Center, Houston, USA); Y. S. Lee (The Catholic University of Korea, Bucheon, Korea); S. Morarasu (Regional Institute of Oncology, Iași, Romania); N. Gouvas (University of Cyprus, Nicosia, Cyprus); T. Zurleni (ASST Fatebenefratelli Sacco, Luigi Sacco Hospital, University of Milan, Milan, Italy); P. Carnevali (ASST Grande Ospedale Metropolitano Niguarda, Milan, Italy); Y. Fukunaga (Cancer Institute Hospital, Japanese Foundation of Cancer Research, Tokyo, Japan); M. Pramateftakis (Aristotle University of Thessaloniki, Thessaloniki, Greece); Z. Balciscueta (Hospital Arnau de Vilanova de Valencia, Valencia, Spain); M. Ali Chaouch (Monastir University Hospital, Monastir, Tunisia); G. J. Chang (The University of Texas, M. D. Anderson Cancer Center, Houston, USA); S. Di Carlo (University of Rome Tor Vergata, Rome, Italy); İ. Hamzaoglu (Acibadem Mehmet Ali Aydinlar University, Istanbul, Turkey); G. Di Buono (University of Palermo, Palermo, Italy); A. Petrov (University Hospitals Dorset, Bournemouth, UK); K. Mathis (Mayo Clinic, Rochester, USA); A. M. Kazaryan (Oslo University Hospital, Oslo, Norway; Østfold Hospital Trust, Grålum, Norway); M. Mazzola (ASST Grande Ospedale Metropolitano Niguarda, Milan, Italy); S. Buscemi (University of Palermo, Palermo, Italy); E. Jovine (Alma Mater Studiorum University of Bologna IRCCS Sant’Orsola, Bologna, Italy); S. Scabini (IRCCS Ospedale Policlinico, San Martino—Genova, Italy); E. Bertani (IEO European Institute of Oncology IRCCS, Milan Italy); G. Ferrari (ASST Grande Ospedale Metropolitano Niguarda, Milan, Italy); J. Khan (University of Portsmouth, Portsmouth, UK); F. Pfeffer (Haukeland University Hospital, Bergen Norway); G. Spinoglio (IRCAD Faculty, IRCAD, Strasbourg, France); G. Luglio (University of Naples Federico II, Naples, Italy); G. Navarra (University of Messina, Messina, Italy); H. Kobayashi (Teikyo University Hospital, Mizonokuchi, Japan); S. Merkel (University Hospital Erlangen, Erlangen, Germany); D. Cassini (Ospedale Città di Sesto San Giovanni, Milan, Italy); G. N. Piozzi (Portsmouth Hospitals University NHS Trust, Portsmouth, UK); M. Spalluto (ASST Ovest Milanese, Legnano, Italy)

## Supplementary Material

zraf066_Supplementary_Data

## Data Availability

Data from the Delphi statements can be obtained upon reasonable request from the corresponding author.

## References

[1] Vogel JD, Felder SI, Bhama AR, Hawkins AT, Langenfeld SJ, Shaffer VO et al The American Society of Colon and Rectal Surgeons clinical practice guidelines for the management of colon cancer. Dis Colon Rectum 2022;65:148–177 34775402 10.1097/DCR.0000000000002323

[2] Argiles G, Tabernero J, Labianca R, Hochhauser D, Salazar R, Iveson T et al Localized colon cancer: ESMO clinical practice guidelines for diagnosis, treatment and follow-up. Ann Oncol 2020;31:1291–1305 32702383 10.1016/j.annonc.2020.06.022

[3] Baxter NN, Kennedy EB, Bergsland E, Berlin J, George TJ, Gill S et al Adjuvant therapy for stage II colon cancer: ASCO guideline update. J Clin Oncol 2022;40:892–910 34936379 10.1200/JCO.21.02538

[4] Le Voyer TE, Sigurdson ER, Hanlon AL, Mayer RJ, Macdonald JS, Catalano PJ et al Colon cancer survival is associated with increasing number of lymph nodes analysed: a secondary survey of intergroup trial INT-0089. J Clin Oncol 2003;21:2912–2919 12885809 10.1200/JCO.2003.05.062

[5] Numata M, Sawazaki S, Aoyama T, Tamagawa H, Sato T, Saeki H et al D3 lymph node dissection reduces recurrence after primary resection for elderly patients with colon cancer. Int J Colorectal Dis 2019;34:621–628 30659360 10.1007/s00384-018-03233-7

[6] Japanese Society for Cancer of the Colon and Rectum . Japanese classification of colorectal, appendiceal, and anal carcinoma: the 3d English edition [secondary publication]. J Anus Rectum Colon 2019;3:175–195 31768468 10.23922/jarc.2019-018 PMC6845287

[7] Heald RJ . The ‘holy plane’ of rectal surgery. J R Soc Med 1988;81:503–508 3184105 10.1177/014107688808100904 PMC1291757

[8] Hohenberger W, Weber K, Matzel K, Papadopoulos T, Merkel S. Standardized surgery for colonic cancer: complete mesocolic excision and central ligation—technical notes and outcome. Colorectal Dis 2009;11:354–364 19016817 10.1111/j.1463-1318.2008.01735.x

[9] Ferri V, Vicente E, Quijano Y, Duran H, Diaz E, Fabra I et al Right-side colectomy with complete mesocolic excision *versus* conventional right-side colectomy in the treatment of colon cancer: a systematic review and meta-analysis. Int J Colorectal Dis 2021;36:1885–1904 33983451 10.1007/s00384-021-03951-5

[10] Anania G, Davies RJ, Bagolini F, Vettoretto N, Randolph J, Cirocchi R et al Right hemicolectomy with complete mesocolic excision is safe, leads to an increased lymph node yield and to increased survival: results of a systematic review and meta-analysis. Tech Coloproctol 2021;25:1099–1113 34120270 10.1007/s10151-021-02471-2 PMC8419145

[11] Sica GS, Vinci D, Siragusa L, Sensi B, Guida AM, Bellato V et al Definition and reporting of lymphadenectomy and complete mesocolic excision for radical right colectomy: a systematic review. Surg Endosc 2023;37:846–861 36097099 10.1007/s00464-022-09548-5 PMC9944740

[12] Dalkey N, Helmer O. An experimental application of the Delphi method to the use of experts. Manage Sci 1963;9:458–467

[13] Page MJ, McKenzie JE, Bossuyt PM, Boutron I, Hoffmann TC, Mulrow CD et al The PRISMA 2020 item: an updated guideline for reporting systematic reviews. BMJ 2021;372:n71 33782057 10.1136/bmj.n71 PMC8005924

[14] Junger S, Payne SA, Brine J, Radbruch L, Brearley SG. Guidance on Conducting and REporting DElphi Studies (CREDES) in palliative care: recommendations based on a methodological systematic review. Palliat Med 2017;31:684–706 28190381 10.1177/0269216317690685

[15] Gattrell WT, Hungin AP, Price A, Winchester CC, Tovey D, Hughes EL et al ACCORD guideline for reporting consensus-based methods in biomedical research and clinical practice: a study protocol. Res Integr Peer Rev 2022;7:3 35672782 10.1186/s41073-022-00122-0 PMC9171734

[16] Nasa P, Jain R, Juneja D. Delphi methodology in healthcare research: how to decide its appropriateness. World J Methodol 2021;11:116–129 34322364 10.5662/wjm.v11.i4.116 PMC8299905

[17] Degiuli M, Resendiz Aguilar AH, Solej M, Azzolina D, Marchiori G, Corcione F et al A randomized phase III trial of complete mesocolic excision compared with conventional surgery for right colon cancer: interim analysis of a Nationwide Multicenter Study of the Italian Society of Surgical Oncology Colorectal Cancer Network (CoME-in trial). Ann Surg Oncol 2024;31:1671–1680 38087139 10.1245/s10434-023-14664-0 PMC10838239

[18] Xu L, Su X, He Z, Zhang C, Lu J, Zhang G et al Short-term outcomes of complete mesocolic excision *versus* D2 dissection in patients undergoing laparoscopic colectomy for right colon cancer (RELARC): a randomized, controlled, phase 3, superiority trial. Lancet Oncol 2021;22:391–401 33587893 10.1016/S1470-2045(20)30685-9

[19] Di Buono G, Buscemi S, Cocorullo G, Sorce V, Amato G, Bonventre G et al Feasibility and safety of laparoscopic complete mesocolic excision (CME) for right-sided colon cancer: short-term outcomes: a randomized clinical study. Ann Surg 2021;274:57–62 33177355 10.1097/SLA.0000000000004557

[20] Tejedor P, Francis N, Jayne D, Hohenberger W, Khan J; CME Project Working Group. Consensus statements on complete mesocolic excision for right-sided colon cancer-technical steps and training implications. Surg Endosc 2022;36:5595–5601 35790593 10.1007/s00464-021-08395-0 PMC9283340

[21] Benz S, Tannapfel A, Tam Y, Grünenwald A, Vollmer S, Stricker I. Proposal of a new classification system for complete mesocolic excision in right-sided colon cancer. Tech Coloproctol 2019;23:251–257 30838463 10.1007/s10151-019-01949-4

[22] Garcia-Granero A, Pellino G, Giner F, Frasson M, Grifo Albalat I, Sánchez-Guillén L et al A proposal for novel standards of histopathology reporting for D3 lymphadenectomy in right colon cancer: the mesocolic sail and superior right colic vein landmarks. Dis Colon Rectum 2020;63:450–460 31996584 10.1097/DCR.0000000000001589

[23] Dindo D, Demartines N, Clavien P-A. Classification of surgical complications: a new proposal with evaluation in a cohort of 6336 patients and results of a survey. Ann Surg 2004;240:205–213 15273542 10.1097/01.sla.0000133083.54934.ae PMC1360123

[24] Amin MB, Edge SB, Greene FL, Byrd DR, Brookland RK, Washington MK et al AJCC Cancer Staging Manual, Eighth Edition. Chicago: American College of Surgeons, 2017

[25] Chen SL, Bilchik AJ. More extensive nodal dissection improves survival for stages I to III of colon cancer: a population-based study. Ann Surg 2006;244:602–610 16998369 10.1097/01.sla.0000237655.11717.50 PMC1856560

[26] Pramateftakis MG . Optimizing colonic cancer surgery: high ligation and complete mesocolic excision during right hemicolectomy. Tech Coloproctol 2010;14:S49–S51 20697925 10.1007/s10151-010-0609-9

[27] Watanabe T, Itabashi M, Shimada Y, Tanaka S, Ito Y, Ajioka Y et al Japanese Society for Cancer of the Colon and Rectum (JSCCR) guidelines 2010 for the treatment of colorectal cancer. Int J Clin Oncol 2012;17:1–29 22002491 10.1007/s10147-011-0315-2

[28] Lu J, Xing J, Zang L, Zhang C, Xu L, Zhang G et al Extent of lymphadenectomy for surgical management of right-sided colon cancer: the randomized phase III RELARC trial. J Clin Oncol 2024;42:3957–3966 39190853 10.1200/JCO.24.00393

[29] Benz SR, Feder IS, Vollmer S, Tam Y, Reinacher-Schick A, Denz R et al Complete mesocolic excision for right colonic cancer: prospective multicentre study. Br J Surg 2022;110:98–105 36369986 10.1093/bjs/znac379 PMC10364501

[30] Garcia-Granero A, Pellino G, Frasson M, Fletcher-Sanfeliu D, Bollina F, Sánchez-Guillén L et al The fusion fascia of Fredet: an important embryological landmark for complete mesocolic excision and D3-lymphadenectomy in right coloncancer. Surg Endosc 2019;33:3842–3850 31140004 10.1007/s00464-019-06869-w

[31] Thorsen Y, Stimec B, Andersen SN, Lindstrom JC, Pfeffer F, Oresland T et al Bowel function and quality of life after superior mesenteric nerve plexus transection in right colectomy with D3 extended mesenterectomy. Tech Coloproctol 2016;20:445–453 27137207 10.1007/s10151-016-1466-y

[32] Piozzi GN, Rusli SM, Baek S-J, Kwak J-M, Kim J, Kim SH. Infrapyloric and gastroepiploic node dissection for hepatic flexure and transverse colon cancer: a systematic review. Eur J Surg Oncol 2022;48:718–726 34893366 10.1016/j.ejso.2021.12.005

[33] Stelzner S, Hohenberger W, Weber K, West NP, Witzigmann H, Wedel T. Anatomy of the transverse colon revisited with respect to complete mesocolic excision and possible pathways of aberrant lymphatic tumor spread. Int J Colorectal Dis 2016;31:377–384 26546443 10.1007/s00384-015-2434-0

[34] Li K, Cao F, He X, Zheng Y. The concept of developmental anatomy: the greater omentum should be resected in right-sided colon cancer? BMC Surg 2023;23:137 37198588 10.1186/s12893-023-02020-8 PMC10193780

