# Supplementary material for: Standardization of the surgical technique and reporting for radical right colectomy with central vascular ligation and complete mesocolic excision (RRoC-STAR): Delphi consensus
Source: BJS Open. 2025 Jun 12;9(3):zraf066. doi: 10.1093/bjsopen/zraf066 (PMC12159727; doi:10.1093/bjsopen/zraf066)
Supplement: zraf066_Supplementary_Data [file zraf066_supplementary_data.docx]

**Standardization of surgical technique and reporting for Radical Right Colectomy with CVL and CME (RROC-STAR): Delphi consensus**

Giuseppe, S. Sica^1^, Gabriele Anania^2^, Cristina Fiorani^1^, Leandro Siragusa^3^, Danilo Vinci^4^, Marco Caricato^5^, Paolo Delrio^6^, Antonino Agrusa^7^, Gianandrea Baldazzi^8^, Rossella Reddavid^9^, Gianluca Pellino^10,11^, for the RRoC-STAR Collaborative Group

^1^ Minimally Invasive and Gastrointestinal Surgery Unit, University of Rome Tor Vergata, Rome, Italy

^2^ Department of Medical Science, University of Ferrara, Ferrara, Italy

^3^ Division of Colon and Rectal Surgery, IRCCS Humanitas Research Hospital, Rozzano, Milan, Italy

^4^ Department of Surgical Science, Policlinico Tor Vergata – University Tor Vergata, Rome, Italy

^5^ Università Campus Bio-Medico di Roma, Rome, Italy

^6^ Colorectal Surgical Oncology, Istituto per lo studio e la cura dei tumori “Fondazione Giovanni Pascale IRCCS”, Naples, Italy

^7^ University of Palermo, Palermo, Italy

^8^ ASST Ovest Milanese, P.O. Nuovo Ospedale di Legnano, Legnano, Italy

^9^ University of Turin, Department of Oncology, San Luigi University Hospital, Italy

^10^ Vall d'Hebron University Hospital, Universitat Autonoma de Barcelona UAB, Barcelona, Spain;

^11^ Department of Advanced Medical and Surgical Sciences Università degli Studi della Campania 'Luigi Vanvitelli', Naples, Italy

**Corresponding author:** Cristina Fiorani, [fiorani.cristina@gmail.com](mailto:fiorani.cristina@gmail.com), orcid [0000-0002-3188-3366](http://orcid.org/0000-0002-3188-3366)

**Supplementary Materials - Index**

| **Supplementary Appendixes** |  |
| --- | --- |
| Appendix 1 | *pag. 2* |
| **Supplementary Figures and Tables** |  |
| Figure S1 | *pag. 3* |
| Figure S2 | *pag. 3* |
| Table S1 | *pag. 4* |
| **References** | *pag. 5* |
|  |  |

**Supplementary Appendixes**

**
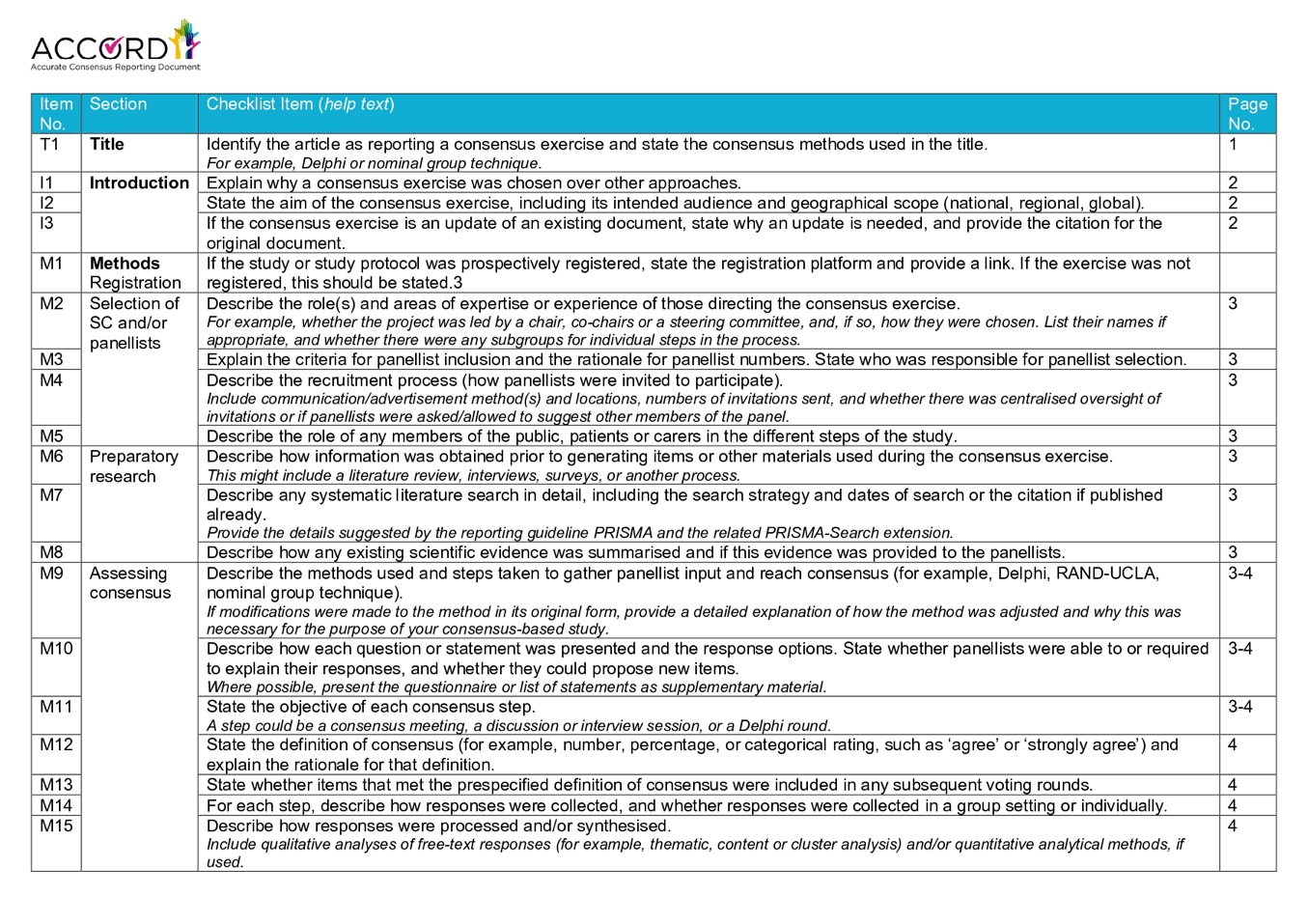
**

**
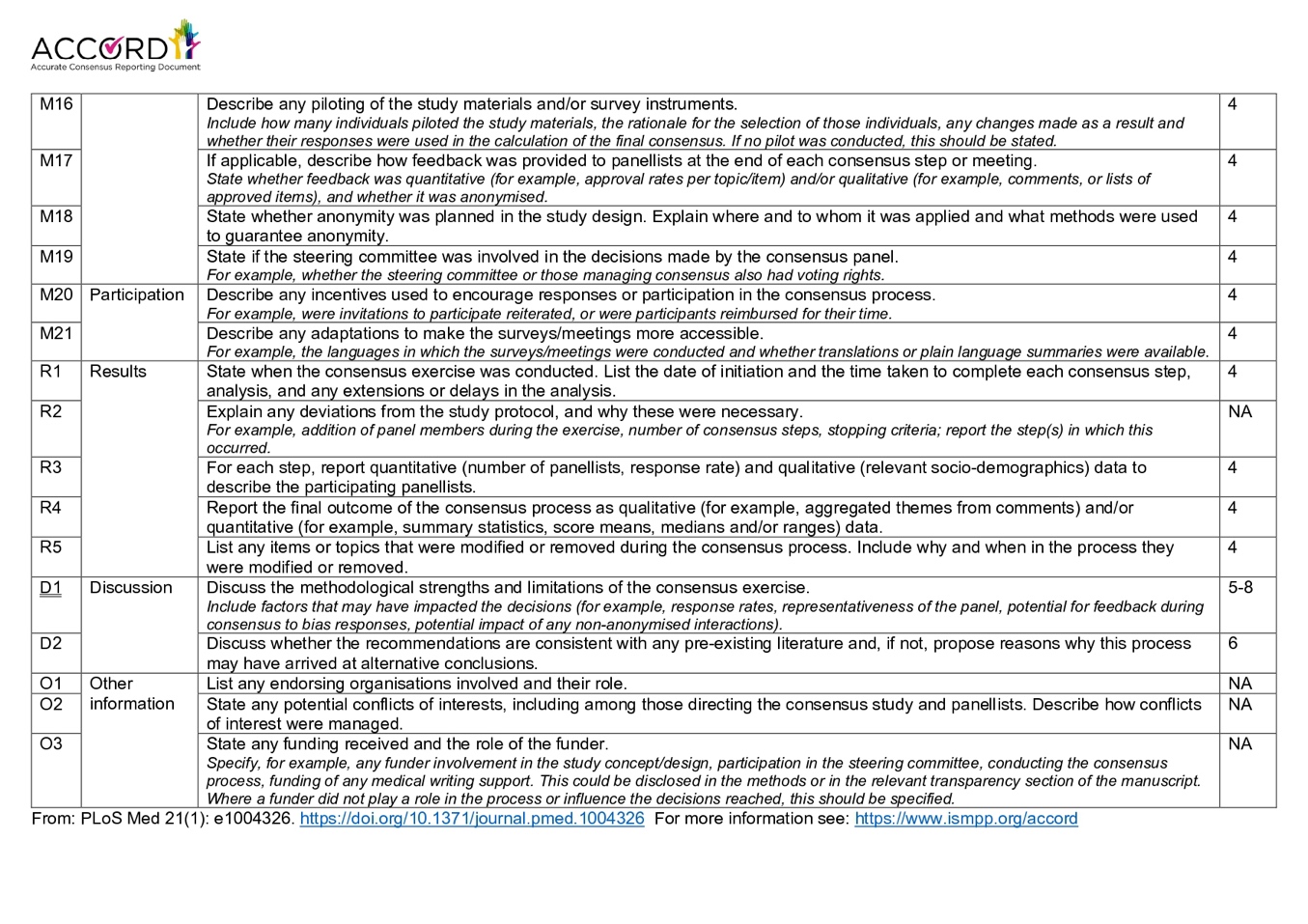
**

**Appendix 1**

**Supplementary Figures and Tables**

**
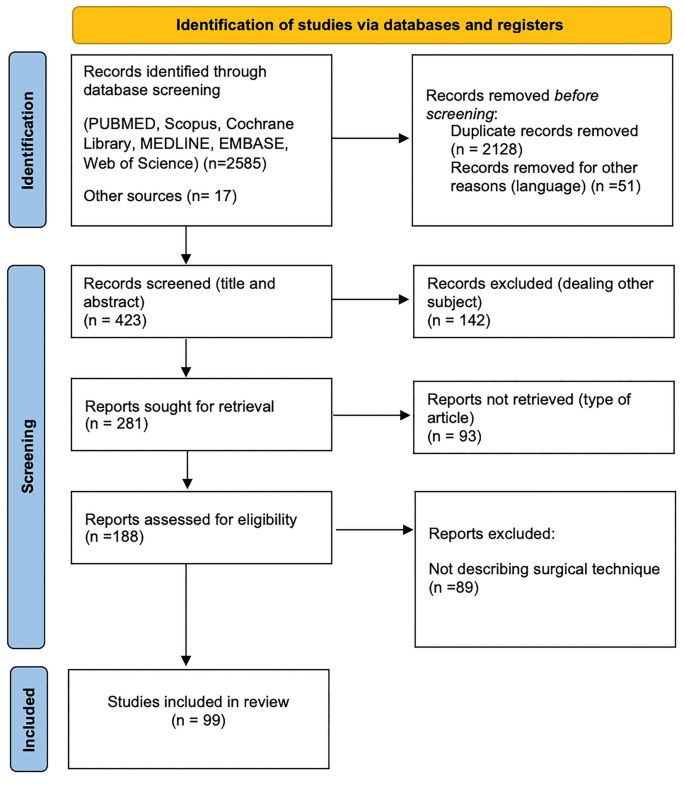
**

**Fig S1**

**
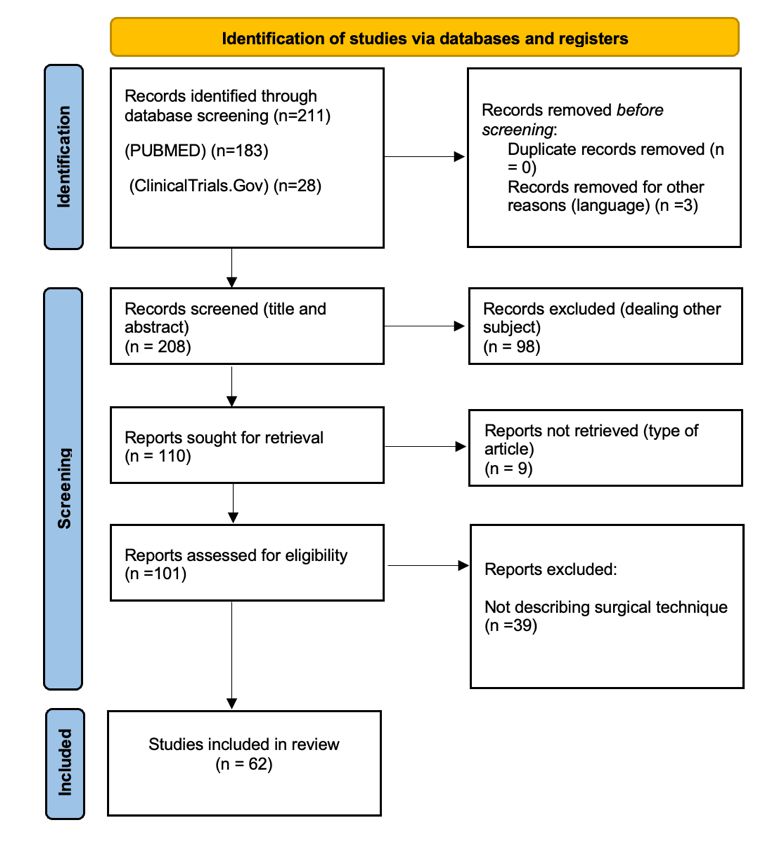
**

**Fig S2**

**
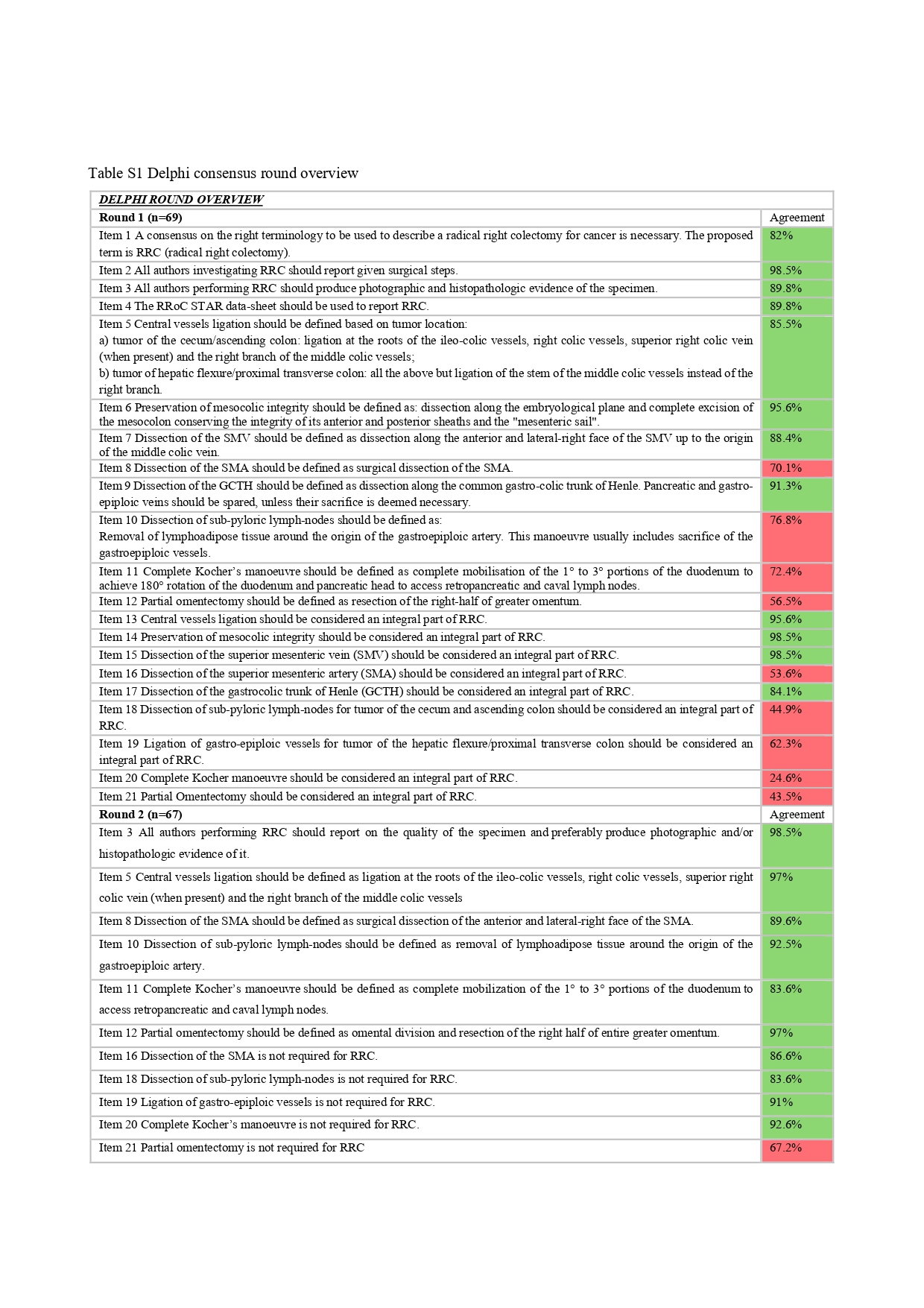
**

**Table S1**

**References**

[1] Vogel JD, Felder SI, Bhama AR, Hawkins AT, Langenfeld SJ, Shaffer VO, Thorsen AJ, Weiser MR, Chang GJ, Lightner AL, Feingold DL, Paquette IM (2022) The American society of colon and rectal surgeons clinical practice guidelines for the management of colon cancer. Dis Colon Rectum 65(2):148–177.

[2] G. Argiles, J. Tabernero, R. Labianca, D. Hochhauser, R. Salazar, T. Iveson, P. Laurent-Puig, P. Quirke, T. Yoshino, J. Taieb, E. Martinelli & D. Arnold. Localised Colon Cancer: ESMO Clinical Practice Guidelines for diagnosis, treatment and follow-up. Ann Oncol 2020 Oct; 31(10):1291-1305.

[3] Nancy N Baxter, Erin B Kennedy, Emily Bergsland, Jordan Berlin, Thomas J George, Sharlene Gill, Philip J Gold, Alex Hantel, Lee Jones, Christopher Lieu, Najjia Mahmoud, Arden M Morris, Erika Ruiz-Garcia, Y Nancy You, Jeffrey A Meyerhardt. Adjuvant Therapy for Stage II Colon Cancer: ASCO Guideline Update. J Clin Oncol. 2022 Mar 10;40(8):892-910.

[4] Voyer TEL, Sigurdson ER, Hanlon AL, et al. Colon cancer survival is associated with increasing number of lymph nodes analysed: a secondary survey of intergroup trial INT-0089. J Clin Oncol. 2003;21: 2912-2919.

[5] Japanese Society for Cancer of the Colon and Rectum (2019) Japanese Classification of colorectal, appendiceal, and anal carcinoma: the 3d English edition [Secondary Publication]. J Anus Rectum Colon 3(4):175–195.

[6] Numata M, Sawazaki S, Aoyama T, Tamagawa H, Sato T, Saeki H, Saigusa Y, Taguri M, Mushiake H, Oshima T, Yukawa N, Shiozawa M, Rino Y, Masuda M (2019) D3 lymph node dissection reduces recurrence after primary resection for elderly patients with colon cancer. Int J Colorectal Dis 34(4):621–628.

[7] Heald RJ. The 'Holy Plane' of rectal surgery. J R Soc Med. 1988 Sep;81(9):503-8.

[8] Hohenberger W, Weber K, Matzel K, Papadopoulos T, Merkel S (2009) Standardized surgery for colonic cancer: complete mesocolic excision and central ligation–technical notes and outcome. Colorectal Dis 11(4):354–364.

[9] Ferri V, Vicente E, Quijano Y, Duran H, Diaz E, Fabra I, Malave L, Agresott R, Isernia R, Cardinal-Fernandez P, Ruiz P, Nola V, de Nobili G, Ielpo B, Caruso R (2021) Right-side colectomy with complete mesocolic excision vs conventional right-side colectomy in the treatment of colon cancer: a systematic review and meta-analysis. Int J Colorectal Dis 36(9):1885–1904.

[10] Anania G, Davies RJ, Bagolini F, Vettoretto N, Randolph J, Cirocchi R, Donini A (2021) Right hemicolectomy with complete mesocolic excision is safe, leads to an increased lymph node yield and to increased survival: results of a systematic review and meta-analysis. Tech Coloproctol 25(10):1099–1113.

[11] Sica GS, Vinci D, Siragusa L, Sensi B, Guida AM, Bellato V, García-Granero Á, Pellino G. Definition and reporting of lymphadenectomy and complete mesocolic excision for radical right colectomy: a systematic review. Surg Endosc. 2023 Feb;37(2):846-861.

[12] Dalkey, N., & Helmer, O. (1963). An Experimental Application of the Delphi Method to the Use of Experts. Management Science, 9(3), 458–467.

[13] Page MJ, McKenzie JE, Bossuyt PM, Boutron I, Hoffmann TC, Mulrow CD et al (2021) The PRISMA 2020 item: an updated guideline for reporting systematic reviews. BMJ 372:n71.

[14] Junger S, Payne SA, Brine J, Radbruch L, Brearley SG. Guidance on Conducting and REporting DElphi Studies (CREDES) in palliative care: recommendations based on a methodological systematic review. Palliat Med. 2017;31:684–706.

[15] Gattrell WT, Hungin AP, Price A, Winchester CC, Tovey D, Hughes EL, et al. ACCORD guideline for reporting consensus-based methods in biomedical research and clinical practice: a study protocol. Res Integr Peer Rev. 2022;7:3.

[16] Nasa P, Jain R, Juneja D. Delphi methodology in healthcare research: How to decide its appropriateness. World J Methodol. 2021 Jul 20;11(4):116-129.

[17] Maurizio Degiuli,Aridai H. Resendiz Aguilar, Mario Solej, Danila Azzolina, Giulia Marchiori, Francesco Corcione, Umberto Bracale, Roberto Peltrini, Maria M. Di Nuzzo, Gianandrea Baldazzi, Diletta Cassini, Giuseppe S. Sica, Brunella Pirozzi, Andrea Muratore, Marcello Calabrò, Elio Jovine, Rafaele Lombardi, Gabriele Anania, Matteo Chiozza, Wanda Petz, Paolo Pizzini, Roberto Persiani, Alberto Biondi, and Rossella Reddavid. A Randomized Phase III Trial of Complete Mesocolic Excision Compared with Conventional Surgery for Right Colon Cancer: Interim Analysis of a Nationwide Multicenter Study of the Italian Society of Surgical Oncology Colorectal Cancer Network (CoME in trial). Ann Surg Oncol 2024 Mar;31(3):1671-1680.

[18] Lai Xu, Xiangqian Su, Zirui He, Chenghai Zhang, Junyang Lu, Guannan Zhang, Yueming Sun, Xiaohui Du, Pan Chi, Ziqiang Wang, Ming Zhong, Aiwen Wu, Anlong Zhu, Fei Li, Jianmin Xu, Liang Kang, Jian Suo, Haijun Deng, Yingjiang Ye, Kefeng Ding, Tao Xu, Zhongtao Zhang, Minhua Zheng, Yi Xiao, on behalf of the RELARC Study Group. Short-term outcomes of complete mesocolic excision versus D2 dissection in patients undergoing laparoscopic colectomy for right colon cancer (RELARC): a randomised, controlled, phase 3, superiority trial. The Lancet Oncology Volume 22, Issue 3, March 2021, Pages 391-401.

[19] Di Buono G, Buscemi S, Cocorullo G, et al. Feasibility and safety of laparoscopic complete mesocolic excision (CME) for right-sided colon cancer: short-term outcomes: a randomized clinical study. Ann Surg. 2021;274:57–62.

[20] Tejedor P, Francis N, Jayne D, Hohenberger W, Khan J; on behalf the CME Project Working Group. Consensus statements on complete mesocolic excision for right-sided colon cancer-technical steps and training implications. Surg Endosc. 2022 Aug;36(8):5595-5601.

[21] Benz S, Tannapfel A, Tam Y, Grünenwald A, Vollmer S, Stricker I. Proposal of a new classification system for complete mesocolic excison in right-sided colon cancer. Tech Coloproctol. 2019 Mar;23(3):251-257.

[22] Garcia-Granero A, Pellino G, Giner F, Frasson M, Grifo Albalat I, Sánchez-Guillén L, Valverde-Navarro AA, Garcia-Granero E. A Proposal for Novel Standards of Histopathology Reporting for D3 Lymphadenectomy in Right Colon Cancer: The Mesocolic Sail and Superior Right Colic Vein Landmarks. Dis Colon Rectum. 2020 Apr;63(4):450-460.

[23] Dindo D, Demartines N, Clavien PA. Classification of surgical complications: a new proposal with evaluation in a cohort of 6336 patients and results of a survey. Ann Surg. 2004 Aug;240(2):205-13.

[24] Amin MB, Edge S, Greene F, et al. AJCC Cancer Staging Manual (8th edition). Springer International Publishing: American J oint Commission on Cancer; 2017.

[25] Chen SL, Bilchik AJ. More extensive nodal dissection improves survival for stages I to III of colon cancer: a population-based study. Ann. Surg. 2006;244:602–610.

[26] Pramateftakis MG. Optimizing colonic cancer surgery: high ligation and complete mesocolic excision during right hemicolectomy. Tech Coloproctol. 2010;14(Suppl 1):S49–S51.

[27] Watanabe T, Itabashi M, Shimada Y et al (2012) Japanese society for cancer of the colon and rectum. japanese society for cancer of the colon and rectum (JSCCR) guidelines 2010 for the treatment of colorectal cancer. Int J Clin Oncol 17:1–29.

[28] Lu J, Xing J, Zang L, Zhang C, Xu L, Zhang G, He Z, Sun Y, Feng Y, Du X, Hu S, Chi P, Huang Y, Wang Z, Zhong M, Wu A, Zhu A, Li F, Xu J, Kang L, Suo J, Deng H, Ye Y, Ding K, Xu T, Zhang Y, Zhang Z, Zheng M, Su X, Xiao Y; RELARC study group. Extent of Lymphadenectomy for Surgical Management of Right-Sided Colon Cancer: The Randomized Phase III RELARC Trial. J Clin Oncol. 2024 Nov 20;42(33):3957-3966. doi: 10.1200/JCO.24.00393. Epub 2024 Aug 27. PMID: 39190853.

[29] Benz SR, Feder IS, Vollmer S, Tam Y, Reinacher-Schick A, Denz R, Hohenberger W, Lippert H, Tannapfel A, Stricker I. Complete mesocolic excision for right colonic cancer: prospective multicentre study. Br J Surg. 2022 Dec 13;110(1):98-105. doi:10.1093/bjs/znac379. PMID: 36369986; PMCID: PMC10364501.

[30] Garcia-Granero A, Pellino G, Frasson M, Fletcher-Sanfeliu D, Bollina F et al. The fusion fascia of Fredet: an important embryological landmark for complete mesocolic excision and D3-lymphadenectomy in right coloncancer. Surgical Endoscopy.

[31] Thorsen Y, Stimec B, Andersen S N, Lindstrom J C, Pfeffer F, Oresland T, Ignjatovic D, RCC study group. Bowel function and quality of life after superior mesenteric nerve plexus transection in right colectomy with D3 extended mesenterectomy. Tech Coloproctol 2016 Jul; 20(7):445-53.

[32] Piozzi GN, Rusli SM, Baek SJ, Kwak JM, Kim J, Kim SH. Infrapyloric and gastroepiploic node dissection for hepatic flexure and transverse colon cancer: A systematic review. Eur J Surg Oncol. 2022 Apr;48(4):718-726.

[33] Stelzner S, Hohenberger W, Weber K, West NP, Witzigmann H, Wedel T. Anatomy of the transverse colon revisited with respect to complete mesocolic excision and possible pathways of aberrant lymphatic tumor spread. Int J Colorectal Dis. 2016 Feb;31(2):377-84. doi: 10.1007/s00384-015-2434-0. Epub 2015 Nov 6.

[34] Li K, Cao F, He X, Zheng Y. The concept of developmental anatomy: the greater omentum should be resected in right-sided colon cancer? BMC Surg. 2023 May 17;23(1):137.
